# Real-World Effectiveness and Predictors of Nurse-Led Individual Cognitive Behavioral Therapy for Mental Disorders: An Updated Pragmatic Retrospective Cohort Study

**DOI:** 10.3390/bs14070604

**Published:** 2024-07-16

**Authors:** Naoki Yoshinaga, Yoko Obara, Naohisa Kawano, Kazuki Kondo, Yuta Hayashi, Michikazu Nakai, Ryuichiro Takeda, Hiroki Tanoue

**Affiliations:** 1 School of Nursing, Faculty of Medicine, University of Miyazaki, 5200 Kihara, Kiyotake, Miyazaki City 889-1692, Miyazaki, Japan; naoki-y@med.miyazaki-u.ac.jp; 2 Graduate School of Nursing Science, University of Miyazaki, 5200 Kihara, Kiyotake, Miyazaki City 889-1692, Miyazaki, Japan; youko_obara@med.miyazaki-u.ac.jp; 3 Cognitive Behavioral Therapy Office, Shigasato Hospital, 1-18-41, Shigasato, Otsu 520-0006, Shiga, Japan; shigasato.hp.cbt@gmail.com; 4 Department of Nursing, Gifu University Hospital, 1-1 Yanagido, Gifu City 501-1194, Gifu, Japan; kondoh.kazuki.h9@f.gifu-u.ac.jp; 5 Department of Nursing, Graduate School of Health Sciences, Kobe University, 7-10-2 Tomogaoka, Suma-ku, Kobe 654-0142, Hyogo, Japan; yuta-h@harbor.kobe-u.ac.jp; 6 Clinical Research Support Center, University of Miyazaki Hospital, 5200 Kihara, Kiyotake, Miyazaki City 889-1692, Miyazaki, Japan; michikazu_nakai@med.miyazaki-u.ac.jp; 7 Health Care and Safety Center, University of Miyazaki, 1-1 Gakuen Kibanadai-Nishi, Miyazaki City 889-2192, Miyazaki, Japan; mhstkd@cc.miyazaki-u.ac.jp

**Keywords:** cognitive behavioural therapy, mental disorders, nurses, retrospective cohort study

## Abstract

The importance of nurses integrating effective psychological techniques into their clinical practice is widely recognized. Nevertheless, further evidence from real-world settings is needed to establish nurse-led cognitive behavioural therapy (CBT) as an effective approach in clinical practice. This study aimed to examine the clinical effectiveness and predictors of individual CBT for mental disorders delivered by nurses in various routine clinical settings. This pragmatic retrospective cohort study collected data from participants who received nurse-led individual CBT at four institutions from different prefectures in Japan between April 2015 and March 2023. During the study period, 280 clients were referred to nurses for CBT, 240 of whom received nurse-led individual CBT of at least one session. The common primary diagnoses among participants were major depressive disorder (33.8%), social phobia (12.9%), and obsessive–compulsive disorder (10.0%). Of these, 23 participants were ongoing cases at the end of the observation period, and 217 who had completed the course of therapy or discontinued/dropped out from the therapy were included in the analysis (173 completed and 44 discontinued/dropped out (i.e., dropout rate = 20.3%)). Based on the clinical significance definition (primary outcome), 62.4% of the participants who completed the therapy were judged to demonstrate positive clinical significance (recovered or improved), with only a few participants (6.9%) demonstrating deterioration. Significant improvements were observed before and after nurse-led individual CBT across all secondary outcomes, including depression and anxiety symptoms, health-related quality of life, and functional disability (all ps ≤ 0.001). Univariate logistic regression revealed that clients with higher baseline severity of depression and anxiety symptoms were less likely to achieve positive clinical significance following nurse-led individual CBT. The real-world evidence gained through this study will encourage frontline nurses and motivate institutional/organizational leaders and policymakers to employ nurse-led individual CBT, especially for depression and anxiety-related disorders.

## 1. Introduction

According to the Global Burden of Disease Study 2019 [1], it is estimated that approximately 970 million people (around one in every eight individuals) worldwide have a mental disorder, with depressive and anxiety disorders being the most prevalent. Cognitive behavioural therapy (CBT) has demonstrated substantial evidence supporting its efficacy across various mental disorders through randomized controlled trials. According to a previous review of meta-analyses [2], robust evidence for the efficacy of CBT was observed, particularly for anxiety disorders, somatoform disorders, bulimia nervosa, and some other mental health problems (anger control problems and general stress). CBT also exhibited higher response rates compared to the comparison conditions, such as other psychological treatments (e.g., relaxation therapy, supportive therapy, or psychodynamic therapy), placebo/control treatments, and usual care [2]. Furthermore, considering the general preference for psychotherapy over pharmacotherapy among individuals with mental disorders [3], it is imperative to continue examining the outcomes of evidence-based psychological therapies (notably CBT) in routine care settings, as well as to improve their accessibility to individuals in need of psychological support.

Efforts to enhance mental health services have underscored the significance of nurses integrating effective psychotherapeutic techniques into their clinical practice [4,5,6]. This does not imply that nurses should act as replacements for psychiatrists or psychologists in delivering psychological therapies; instead, they should be considered autonomous healthcare professionals with a holistic, eclectic, and relational approach [4,5,6]. Nevertheless, as nurses form the largest occupational group in the global health workforce, they are also expected to play a significant role in disseminating evidence-based psychological therapies in clinical practice. For example, in Japan, CBT provided by psychiatrists was initially incorporated into the national health insurance scheme in 2010, marking a milestone in Japanese psychiatric services. Subsequently, in 2016, the eligibility for CBT providers was extended to include nurses in order to make CBT more available [7,8].

The efficacy of nurse-led CBT for mental disorders has also been demonstrated through several randomized controlled trials [9,10,11,12]; however, global concerns have been raised regarding the generalizability of the results of efficacy studies (i.e., external validity), particularly highly selective clinical trials, to real-world clinical practices. Efficacy studies are often conducted in highly controlled settings, employing strict inclusion and exclusion criteria, such as the exclusion of clients with comorbid diagnoses or those receiving concurrent medications, or the establishment of symptom severity thresholds. In fact, a significant portion of clients seeking treatment are often excluded from efficacy studies because of such strict selection criteria, and those who do meet the criteria and agree to randomization may not be representative of typical clients seeking treatment in daily practice [13,14]. Interventions are also controlled in efficacy studies, with study therapists often required to strictly adhere to the CBT protocol and receive more intensive training and supervision compared to community therapists. The existence of pragmatic randomized controlled trials, which are designed to be more closely aligned with routine clinical settings, offers some promise in bridging the research–practice gap; however, challenges persist in accurately reflecting the complexities of real-world clinical practice [12,15]. Thus, treatments/interventions should be evaluated from both an efficacy and effectiveness perspective.

To evaluate the external validity of empirically-supported psychological therapies (i.e., assessing “effectiveness”), it is useful to consider their clinical representativeness, which is achieved through the following characteristics [13,16,17,18]: including nonrandomized clients who are typical referrals to routine clinical settings outside of academic settings, having therapists delivering treatment in hospitals/clinics, absence of treatment implementation monitoring or therapist training/supervision for study purposes, and adopting an open-ended, flexible structure of treatment. Some researchers have also argued that a prospective study requiring clients to sign an informed consent form in order to participate (e.g., pragmatic randomized controlled trial, prospective cohort study) might be less pragmatic because client awareness of study participation can influence outcomes due to the Hawthorne effect [19,20]. Employing a retrospective study design that analyzes the client outcomes of interventions already archived in routine clinical practice is one possible approach to address these limitations and enhance external validity (clinical representativeness). Within this context, findings from studies retrospectively analyzing routine outcome data collected within the Improving Access to Psychological Therapies (IAPT) services in the UK (now renamed as NHS Talking Therapies) have provided substantial real-world evidence of the effectiveness of psychological therapies for mental disorders [21]. The UK-IAPT services systematically collect routine outcome data from almost all individuals undergoing therapy via a session-by-session outcome monitoring system, and the results of local and national analyses using the collected data have been reported. A previous study conducted a meta-analysis of 10 years of practice-based evidence arising from UK-IAPT services, demonstrating that CBT and other psychological therapies provided by multiprofessionals yielded large pre–post treatment effect sizes for depression and anxiety symptoms, with a medium effect size for work and social adjustment [22]. The UK-IAPT systems have subsequently been adopted in some other countries (e.g., Australia and Norway), providing similar evidence of the clinical effectiveness of CBT [23,24]. This accumulating real-world evidence on CBT, which previously was primarily confined to small-scale and tightly controlled studies, helps bridge the efficacy–effectiveness (research–practice) gap. However, almost all of the studies utilizing routine outcome data on CBT provided by multiprofessionals did not report outcome results by profession, resulting in limited knowledge about the clinical effectiveness of CBT provided by nurses.

Therefore, we previously conducted a retrospective chart review of outpatients with mental disorders who underwent individual CBT provided by nurses in routine psychiatric outpatient care settings [25]. The results revealed significant improvements in clients’ depressive and anxiety symptoms, as well as their subjective quality of life, before and after nurse-led CBT. Furthermore, these improvements were sustained among those who received optional follow-up. Our earlier study, therefore, successfully provided initial evidence of the effectiveness of nurse-led CBT in routine care settings. To the best of our knowledge, no studies outside Japan have investigated such routine clinical outcomes of nurse-led individual CBT for mental disorders. However, this earlier study was constrained by its small sample size (n = 100), its focus solely on outpatients, and data collection from institutions located only in Miyazaki prefecture. These limitations restrict the robustness and generalizability of the findings to wider routine psychiatric care practices in Japan [25].

In addition to evaluating the clinical effectiveness of CBT, gaining a deeper understanding of “what works for whom” is crucial for advancing towards more personalized care, which may potentially enhance treatment outcomes and optimize the allocation of limited mental healthcare resources. While various studies using routinely collected data in UK-IAPT services have explored and documented several predictors of post-treatment outcomes [21], our earlier study was hampered by limited sample size (n = 100) [25], which prevented us from conducting a predictor analysis.

Taking into account the limitations identified in our earlier study [25], the present study aimed to further examine the clinical effectiveness and baseline predictors of nurse-led individual CBT for mental disorders in routine psychiatric care settings, based on a larger sample than our earlier study. To achieve this, we collected data from clients who received nurse-led individual CBT by extending coverage to hospitals or clinics beyond Miyazaki prefecture and including a wider range of clinical settings beyond outpatient care.

## 2. Materials and Methods

We report this study in accordance with the Strengthening the Reporting of Observational Studies in Epidemiology (STROBE) guidelines [26].

### 2.1. Study Design

This study employed a retrospective cohort design.

### 2.2. Setting

We collected routine outcome data from clients with mental disorders who received individual CBT from nurses at four study institutions in Japan between April 2015 and March 2023. Study institutions were selected through the researchers’ networks; they were from Miyazaki prefecture (one psychiatric clinic and one psychiatric hospital), Shiga prefecture (one psychiatric hospital), and Gifu prefecture (one university hospital). The Research Ethics Committee of the Faculty of Medicine, University of Miyazaki, approved the study protocol (approval number: O-1314), and granted a waiver of obtaining informed consent from each client due to the study’s retrospective nature (i.e., employing an opt-out method in which potential participants were given the opportunity to refuse study participation).

### 2.3. Participants

The inclusion criteria were as follows: (1) the client had a primary diagnosis of mental disorders (codes F00–F99 and G47) listed in the 10th edition of the International Classification of Diseases (ICD-10); (2) was aged ≥ 20 years at the initial assessment with a nurse; and (3) had received nurse-led individual CBT of at least one session between April 2015 and March 2023. The diagnostic assessment was performed by a primary psychiatrist at the study institution.

### 2.4. Intervention

Individual CBT was delivered by five nurses at the study institutions. Their background information was separately collected by asking each nurse to fill out an Excel sheet. On average, they had clinical experience of 14.5 years (min: 9.8, max: 20.9) in psychiatric nursing and 8.7 years (min: 7.0, max: 10.5) in providing CBT. All nurses had completed a formal, multiprofessional CBT training program in Japan (including case supervision), which is organized by the Japanese Ministry of Health, Labour and Welfare. Two out of five nurses were Certified Behavioural Therapists of the Japanese Association of Behavioural and Cognitive Therapies, and one was a Certified Cognitive Therapist of the Academy of Cognitive and Behavioural Therapies.

Nurses provided individual CBT through a structured therapy session containing components of cognitive and/or behavioural techniques in different routine clinical settings. For mood disorders, social anxiety disorder (social phobia), panic disorder, obsessive–compulsive disorder, post-traumatic stress disorder, and bulimia nervosa, nurses used standardized CBT protocols/manuals approved by the national health insurance scheme in Japan [27,28]. Most sessions lasted approximately 30–60 min.

### 2.5. Clinical Outcomes

Routine clinical outcome data were collected at baseline and at the end of CBT; for participants who discontinued or dropped out from the therapy, outcome data at baseline and at the last therapy session (i.e., last observed outcome data) were collected.

Collected self-reported clinical outcome data were as follows: (1) the 9-item Patients Health Questionnaire (PHQ-9) [29,30,31] for assessing the severity of depressive symptoms, (2) the 7-item Generalized Anxiety Disorder scale (GAD-7) [32,33] for assessing the severity of anxiety symptoms, (3) the EuroQoL 5-dimension 5-level (EQ-5D-5L) [34,35,36] for assessing health-related quality of life, and (4) the Sheehan Disability Scale (SDS) [37,38] for assessing functional disability. However, since the outcome data used in this study were collected within real-world clinical practice in each institution, not all institutions routinely collected all these outcome measures (except for PHQ-9 and GAD-7): EQ-5D-5L was collected at two institutions, and SDS was collected at the remaining two institutions.

We set the clinical significance as a primary outcome, which refers to clinically meaningful changes for individuals as they progress through a course of CBT. Based on the established reliable change indices and cut-off points of the PHQ-9 and GAD-7 [39], participants were classified as “Recovered”, “Improved”, “No reliable change (unproblematic or problematic)”, or “Deteriorated” (Table 1). All the other outcome measures (PHQ-9, GAD-7, ED-5D-5L, and SDS) were considered secondary outcomes.

### 2.6. Sample Size

When developing a prediction model, logistic regression analysis should be used with at least 10 events per variable, and is acceptable with 5–9 events per variable [40,41,42,43]. In this study, we estimated that 50% of participants would demonstrate positive clinical significance following nurse-led individual CBT based on our earlier study [25], and selected 12 potential predictors (described in more detail in the analysis section). To achieve an event per variable ratio of 8 for our logistic regression model, we required a sample size of at least 192 participants (the event per variable ratio [“96 (50%) participants demonstrating positive clinical significance” divided by “12 potential predictors”] = 8).

### 2.7. Analysis

Descriptive statistics were provided using means and standard deviations (SDs) for continuous variables and frequencies and proportions for categorical data to present the baseline characteristics of the sample and clinical significance (primary outcome).

We used paired t-tests to compare the average scores for each secondary outcome measure between baseline and the end of CBT. The magnitude of the intervention effect was determined as the within-group pre–post effect size based on Hedges’ g; an effect size of <0.20 is interpreted as a negligible effect, 0.20–0.49 as a small effect, 0.50–0.79 as a moderate effect, and ≥0.80 as a large effect.

We performed a primary analysis based on data obtained from participants at the baseline and at the end of CBT timepoints without imputing missing data. To examine the effects of missing data, a sensitivity analysis was performed by imputing missing data where the last observed scores for participants who discontinued or dropped out were carried forward (i.e., intention-to-treat analysis). To examine the confounding effects of concurrent psychotropic medications, we compared subgroup results of participants who received or did not receive concurrent psychotropic medications at baseline using Welch’s t-Test.

To examine the predictors of positive clinical significance status (“Recovered” or “Improved”) following nurse-led individual CBT, unadjusted associations between positive clinical significance status as a dependent variable and each of the possible predictors as independent variables were analyzed using univariate logistic regression. The associations between the independent and dependent variables were presented as odds ratios (ORs) with 95% confidence intervals (CIs). Twelve possible predictor variables were selected: (1) age (continuous), (2) gender (female or male/gender neutral), (3) marital status (having or not having a partner), (4) employment status (employed or unemployed), (5) primary diagnosis (“mood [affective] disorders”, “neurotic, stress-related, and somatoform disorders”, “disorders of psychological development”, “schizophrenia, schizotypal, and delusional disorders”, “mental and behavioural disorders due to psychoactive substance use”, or “others”), (6) duration of primary diagnosis (continuous), (7) comorbidity (yes or no), (8) baseline antidepressant dose (continuous), (9) baseline anxiolytic dose (continuous), (10) baseline PHQ-9 score (continuous), (11) baseline GAD-7 score (continuous), and (12) study institution (institution I, II, III, or IV).

Data analysis was performed using JMP Pro Version 16.1.0 (SAS Institute Japan, Tokyo, Japan), and a two-tailed alpha level of 0.05 was used to define statistical significance.

## 3. Results

### 3.1. Participant Flow

**Figure 1:**
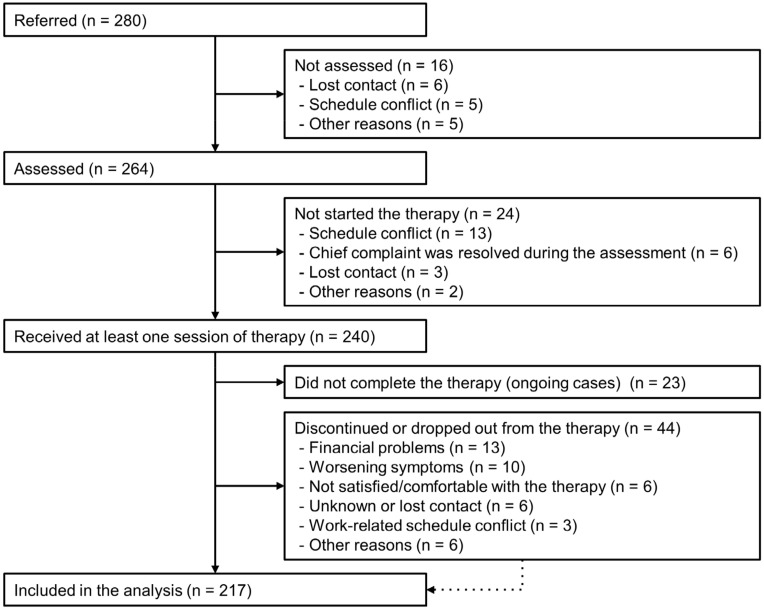
Flow diagram of participants.

During the study period, 280 clients were referred to nurses for CBT, 40 of whom were excluded. The remaining 240 clients received nurse-led individual CBT of at least one session. Of these, 23 participants were ongoing cases at the end of the observation period, and 217 who had completed the course of therapy (planned CBT sessions) or discontinued/dropped out from the therapy were included in the analysis (173 completed and 44 discontinued/dropped out) (Figure 1). Some participants discontinued the therapy for positive reasons (e.g., moved to a different city, or work-related schedule conflict occurred as they successfully started or returned to work during the therapy).

### 3.2. Baseline Sociodemographic and Clinical Characteristics

Table 2 shows the participants’ baseline sociodemographic and clinical characteristics. Among the 240 participants who received at least one session of individual CBT by a nurse, the mean age was 36.6 years (SD = 12.3), and 119 participants (49.6%) were female. The most common primary diagnosis was major depressive disorder (n = 81 (33.8%)), followed by social phobia (n = 31 (12.9%)) and obsessive–compulsive disorder (n = 24 (10.0%)). The mean duration of primary diagnosis was 7.6 years (SD = 7.7), and 90 participants (37.5%) had comorbid mental disorders. In addition, 157 participants (72.9%) received concurrent psychotropic medications at baseline.

### 3.3. Intervention Received

Among the 240 participants who received at least one session of nurse-led individual CBT, the most common therapy setting was outpatient (n = 227 (94.5%)); the other settings were inpatient (n = 4 (1.7%)) and a combination of inpatient and outpatient (n = 9 (3.8%)). Each nurse handled an average of 48 cases (min: 15, max: 82) during the study period. Among the 173 participants who completed the course of therapy (dropout rate = 20.3% (44/217)), on average, 13.6 sessions (SD = 7.0) were conducted over 35.5 weeks (SD = 30.0).

### 3.4. Clinical Outcomes

Table 3 presents the clinical significance after receiving nurse-led individual CBT (primary outcome). Among the 173 completer sample (primary analysis), 74 participants (42.8%) were classified as “Recovered”, and 34 (19.7%) as “Improved” (i.e., 108 (62.4%) participants demonstrated positive clinical significance). The remaining 65 participants (37.6%) were judged to have “No reliable change (unproblematic/problematic)” or “Deteriorated”. In the sensitivity analysis where missing data were imputed (n = 217), 120 participants (55.3%) met the criteria for positive clinical significance, and 97 participants (44.7%) were classified as “No reliable change (unproblematic/problematic)” or “Deteriorated”. In the subgroup analysis by baseline concurrent psychotropic medications status, there was no significant difference in the proportion of clinical significance between subgroups.

Table 4 presents the results of secondary outcome measures (PHQ-9, GAD-7, EQ-5D-5L, and SDS). In the primary analysis (n = 173), statistically significant improvements were found in all secondary outcome measures (all ps ≤ 0.001). The within-group effect sizes for the PHQ-9 and GAD-7 were large (Hedges’ g = 0.84 and 0.88, respectively), and those for EQ-5D-5L and SDS were medium (Hedges’ g = 0.71 and 0.51, respectively). In the sensitivity analysis (n = 217), statistically significant improvements were replicated in all secondary outcome measures (all ps ≤ 0.001), but the effect sizes were smaller than those observed in the primary analysis. We confirmed that there was no significant difference in pre–post changes in these secondary outcomes between subgroups (with or without concurrent psychotropic medications at baseline).

### 3.5. Predictors of Positive Clinical Significance

Among the 12 possible predictors, two variables (baseline PHQ-9 and GAD-7 scores) were found to be significant in univariate logistic regression (Table 5). More specifically, the chances of positive clinical significance following nurse-led individual CBT decrease by approximately 17% with an increase of one point on the baseline PHQ-9 score (OR = 0.83, 95% CI = 0.78–0.89), and decrease by approximately 22% with an increase of one point on the baseline GAD-7 score (OR = 0.78, 95% CI = 0.72–0.85).

**Figure 2:**
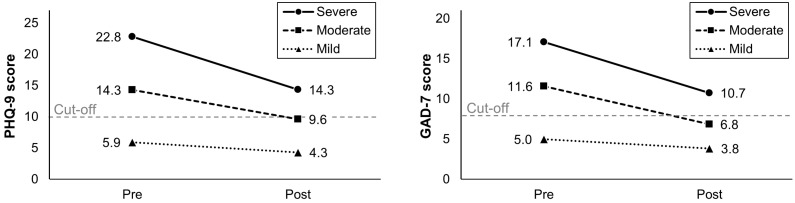
Comparison of PHQ-9 and GAD-7 scores before and after nurse-led individual CBT by baseline severity groups (n = 217, excluding ongoing cases).

**Figure A1:**
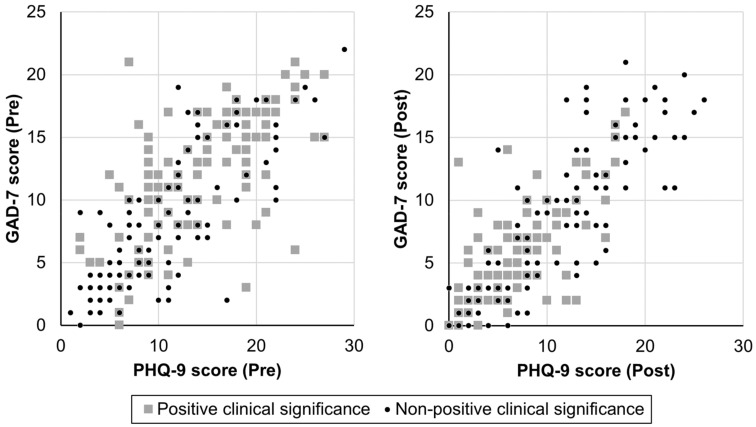
Scatter plot between PHQ-9 and GAD-7 scores before and after nurse-led individual CBT stratified by clinical significance (n = 217, excluding ongoing cases).

As a post hoc analysis, we examined the correlation between the two predictors (PHQ-9 and GAD-7 scores) before and after nurse-led CBT, stratified by clinical significance (positive and nonpositive clinical significance). The results demonstrated correlation coefficients of at least 0.63 in both pre- and post-CBT timepoints, indicating a moderate to high level of association between the two predictors (see Figure A1 and Table A1 in Appendix A). Furthermore, to investigate whether participants with higher baseline scores on PHQ-9 and GAD-7 benefited from nurse-led CBT, we compared pre–post changes in each score by categorizing participants into three baseline severity groups based on established norms (mild, moderate, or severe) (Figure 2). Using a Kruskal–Wallis test, we found that increasing baseline severity was associated with greater improvement in both PHQ-9 and GAD-7 scores (both ps < 0.001), with the greatest improvement observed in participants who started nurse-led CBT with severe symptoms (pre–post effect sizes: 1.75 for PHQ-9 and 1.55 for GAD-7 in the severe group, 1.07 for PHQ-9 and 1.47 for GAD-7 in the moderate group, and 0.48 for PHQ-9 and 0.36 for GAD-7 in the mild group).

## 4. Discussion

This study aimed to re-examine the clinical effectiveness and predictors of nurse-led individual CBT for mental disorders in various routine clinical settings. Compared to our earlier study (n = 100) [25], we successfully increased the number of study institutions from different prefectures and the number of participants included in the analysis (n = 217, excluding ongoing cases); but the main clinical settings were still predominantly outpatient. The results of this updated study yielded three key findings. First, more than half of the participants were judged to have demonstrated positive clinical significance (recovered or improved) through receiving nurse-led individual CBT, with only a few participants deteriorating. Second, nurse-led individual CBT led to significant improvements in depression and anxiety symptoms, health-related quality of life, and functional disability. Third, clients with higher baseline severity of depression and anxiety symptoms had a lower likelihood of achieving positive clinical significance following nurse-led individual CBT.

The event per variable ratio (“108 participants demonstrating positive clinical significance” divided by “12 potential predictors”) was 9 in our logistic regression model. This suggests that our study had a sufficient sample size for the predictor analysis, meeting the recommended acceptable range of 5–9 events per variable [40,41,42,43].

### 4.1. Sample Characteristics Compared to Our Earlier Study

Baseline sample characteristics in this study were largely similar to those in our earlier study [25], such as age, gender, marital status, employment status, and comorbidity. However, there are slight differences in the proportion of primary diagnosis and participants who received concurrent psychotropic medications at baseline between these studies. The top two major primary diagnoses were major depressive disorder and social phobia (social anxiety disorder) in both studies, but the proportion of obsessive–compulsive disorder increased (earlier study: 4%, current study: 15%). It is unclear how one should interpret this difference, but it may be because two out of the five nurses in this study (who were not included in our earlier study) were Certified Behavioural Therapists specializing in exposure and response prevention therapy, which is commonly used for the treatment of obsessive–compulsive disorder (they provided CBT for 11 of 24 (52.4%) cases with obsessive–compulsive disorder in this study). Furthermore, the proportion of participants who received concurrent psychotropic medications at baseline increased from our earlier study (earlier study: 62.0%, current study: 72.9%). The reason for this is also unclear, but our subgroup analysis confirmed that this confounding factor (the baseline concurrent psychotropic medication status) did not affect intervention outcomes.

### 4.2. Real-World Effectiveness Compared to Our Earlier Study and Studies in Other Countries

Similar to our earlier study [25], over half of the participants demonstrated positive clinical significance following nurse-led CBT (earlier study: 56.0%, current study: 55.3%, both based on intention-to-treat sample). As for pre–post effect sizes in secondary outcomes based on intention-to-treat sample, effect sizes on depression and anxiety symptom severities (PHQ-9 and GAD-7) were moderate in both studies, but those on subjective quality of life (EQ-5D-5L) increased (earlier study: small (0.40), current study: moderate (0.53)). The dropout rate was also slightly improved (earlier study: 25.0%, current study: 20.3%). We cannot clearly explain why these differences exist. One possible explanation is that three out of the five nurses in this study were also included in our earlier study, so their further clinical experience in providing CBT after our earlier study might have contributed to improving intervention outcomes. However, there are mixed findings on the influence of therapists’ clinical experience in CBT on client outcomes provided [44,45]. To summarize, our findings from this study with larger samples replicated those observed in our earlier study for the most part [25], providing further real-world evidence of the effectiveness of individual CBT for mental disorders delivered by nurses.

To the best of our knowledge, there is no study outside Japan investigating routine clinical outcomes of nurse-led CBT, but several studies have reported routine CBT outcomes for mental disorders provided by multiprofessionals (primarily clinical psychologists). For example, studies using routine outcome data within the Improving Access to Psychological Therapies (IAPT) services in the UK reported that 30–50% of clients achieved “Recovered” criteria after receiving CBT (mostly high-intensity therapy) [46,47,48,49,50]. Studies from UK-IAPT, Australian IAPT, and German clinical practice demonstrated that within-group pre–post effect sizes of CBT (mostly high-intensity therapy) were 0.63–1.19 on symptom severity (e.g., PHQ-9, GAD-7, Beck Depression Inventory, and Brief-Symptom Inventory) [22,23,46,50,51]. It is difficult to directly compare these results with those in our study. However, considering that a recent meta-analysis evaluating the effectiveness of psychological interventions (including CBT) in routine practice demonstrated that Asian samples had smaller effect sizes compared to UK samples [52], our results in terms of clinical significance (“Recovered” = 36.4% based on intent-to-treat analysis) and pre–post effect sizes (0.66–0.67 on PHQ9/GAD-7 based on intent-to-treat sample) were largely comparable to those observed in routine clinical settings from other countries.

### 4.3. Predictor of Intervention Outcome

Several studies using UK-IAPT data have identified several baseline predictors of CBT outcomes (e.g., reliable recovery and/or improvement) for mental disorders in routine clinical practice [49,53,54,55]. Among the possible predictor variables (or similar variables) included in our analysis, several studies reported similar baseline predictors that high baseline symptomatic severity of depression and/or anxiety had been associated with poor treatment outcomes [49,53,54,55], whereas other predictors (that are not significant in our study) were also associated in some studies, such as age (younger age), employment status (unemployed active job seekers and long-term sick/disabled), and functional disability (higher functional impairment) [54,55]. It is not surprising that this study and other studies similarly identified baseline symptom severity as a predictor of client post-intervention outcomes [49,53,54,55]. In order to be classified as having positive clinical significance following CBT based on its definition in our study and UK-IAPT, clients with higher baseline symptom severity need to show considerably more symptomatic improvement than those with lower baseline symptom severity [54].

It should also be noted that our results from predictor analysis do not imply that clients with more severe baseline symptoms benefited less from nurse-led CBT than those with less severe baseline symptoms. The observation of symptomatic improvements across all levels of baseline symptomatic severity in our post hoc analysis suggests that CBT provided by experienced nurses benefits clients over the full range of severity. Furthermore, clients with severe symptoms demonstrated considerably greater improvement than those with moderate and mild symptoms. These findings largely align with those from previous UK studies [49,54]. Nevertheless, although clients with more severe baseline symptoms are expected to demonstrate greater symptomatic improvements following nurse-led CBT, they are also less likely to score below the clinical cut-off on the PHQ-9 and/or the GAD-7 at the completion of the course of therapy. This can be explained by the fact that starting CBT with higher scores on the PHQ-9 and/or GAD-7 offers greater potential for improvement; however, it also indicates a greater distance from the clinical cut-off points, thereby making it difficult to reach the criteria for positive clinical significance. This suggests that nurses should anticipate in advance that clients presenting with more severe symptoms during the initial assessment may require longer-term intervention and follow-up to fully recover from their illness. For example, nurses could perhaps have some flexibility in session schedules for such clients by not filling all available session time slots or by refraining from scheduling new client appointments in the same time slots immediately after the completion of planned therapy sessions for such clients.

### 4.4. Limitations

Findings should be considered in light of the following limitations. First, our study purposefully employed a retrospective study design, but this does not allow for controlling potential confounding factors that might have contributed to the observed participant outcomes. As this study was conducted within a routine care setting, this was an unavoidable limitation. Second, this study had no comparison group because clients who received treatment/intervention other than CBT at study institutions were not routinely assessed using the same validated self-reported questionnaires (e.g., PHQ-9 and GAD-7). Third, this updated study still had only a few nurses providing CBT, mainly due to the limited number of nurses and institutions routinely providing CBT and collecting core clinical outcomes (e.g., PHQ-9 and GAD-7 recommended by the UK- and Australian IAPT, and the Japanese Society of Anxiety and Related Disorders).

Further studies should replicate the findings and address the limitations of this study. To achieve this, a core outcome measurement set (e.g., PHQ-9 and GAD-7) should be standardized and utilized in wider routine clinical practice. This would facilitate the accumulation of further real-world data on nurse-led individual CBT, allowing comparisons at local, national, and international levels. Conducting a prospective cohort or case–control study is also worth considering, as a prospective design may overcome some of the limitations of a retrospective design: the available data may be of poor quality as they were not designed/standardized for the study in advance, and there is frequently an absence of data on potential confounding factors. Furthermore, while the primary focus of this study lies on nurse-led individual CBT for clients with primary diagnoses of mental disorders within psychiatric care settings, it is also crucial for future research to focus on clients with physical health problems who also experience mental health issues (e.g., depression and anxiety symptoms in clients with cancer, heart failure, respiratory diseases, and chronic pain).

### 4.5. Future Implications

It should be noted that the nurses who provided CBT in our study had sufficient experience in psychiatric nursing (over 9.8 years) and in providing CBT (over 7.0 years). As stated, although there is no clear evidence of whether there is an association between therapists’ clinical experience in CBT and client outcomes [44,45], it means that findings from this study provide real-world evidence of the effectiveness of individual CBT when delivered by “experienced nurses”. This evidence, however, suggests that Japan’s government-led CBT training programme and other opportunities (workshops at academic conferences) work effectively in producing competent therapists because all the nurses have completed this training programme. Thus, it is hoped that the real-world evidence of experienced nurse-led CBT revealed through this study will inspire frontline nurses to receive such training programs, encourage institutional/organizational leaders to support more nurses in receiving CBT training, and motivate policymakers to invest more in training nurses in CBT. The limited generalizability of the study findings (i.e., CBT was delivered only by experienced nurses) also suggests that more work is needed towards wide-scale dissemination of CBT across the country. Since many studies indicated the limited time availability in providing CBT among nurses [56,57,58], it is necessary to develop interventions and training tailored to nurses’ readiness and skill level in CBT (e.g., simple and flexible CBT interventions that can be used by different levels of nurses and in different care settings) [59,60].

## 5. Conclusions

Our findings through this updated pragmatic retrospective cohort study suggest that individual CBT provided by nurses in routine psychiatric care settings is effective for individuals with mental disorders. This is evidenced by the observed excellent clinical outcomes, including the large proportion of participants classified as positive clinical significance and medium to large pre–post effect sizes in all outcome measures. We also identified baseline predictors of nurse-led CBT outcomes in which higher baseline severity of depression and anxiety symptoms are associated with a lower likelihood of achieving positive clinical significance following nurse-led individual CBT. The real-world evidence gained through this study will encourage frontline nurses and motivate institutional/organizational leaders and policymakers to employ nurse-led CBT, especially for depression and anxiety-related disorders.

## Figures and Tables

**Table 1:** The definition of clinical significance (primary outcome).

Category	Definition
**“Recovered”**	Individuals who reliably improved (PHQ-9 reduction of ≥5 points and/or GAD-7 reduction of ≥ 4 points from baseline) and whose last observed scores were below the cut-off point (PHQ-9 < 10 and GAD-7 < 8)
**“Improved”**	Individuals who reliably improved but whose last observed scores were above the cut-off point (PHQ-9 ≥ 10 or GAD-7 ≥ 8)
**“No reliable change (unproblematic)”**	Individuals who showed no reliable change but whose last observed scores were below the cut-off point
**“No reliable change (problematic)”**	Individuals who showed no reliable change and whose last observed scores were above the cut-off point
**“Deteriorated”**	Individuals who showed a reliable change in the opposite direction (deterioration of ≥5 points on PHQ-9 and/or ≥4 points on GAD-7 from baseline)

Abbreviations: GAD-7, the 7-item Generalized Anxiety Disorder scale; PHQ-9, the 9-item Patients Health Questionnaire.

**Table 2:** Baseline sociodemographic and clinical characteristics of the participants who received at least one session of nurse-led individual CBT (n = 240).

Variable		Value
**Age**, years, mean (SD)		36.6 (12.3)
**Gender**, n (%)		
Female		119 (49.6)
Male or gender neutral		121 (50.4)
**Marital status**, n (%)		
Having a partner	Married or living as married	82 (34.2)
Not having a partner	Single or never married	137 (57.1)
	Others (e.g., divorced)	6 (2.5)
	*[total]*	*[143 (59.6)]*
**Employment status**, n (%)		
Employed	Full-time employment	69 (28.8)
	Part-time employment	32 (13.3)
	*[total]*	*[101 (42.1)]*
Unemployed	Student	23 (9.6)
	Sick leave from work or school	40 (16.6)
	Unemployed or homemaker	76 (31.6)
	*[total]*	*[143 (59.6)]*
**Primary diagnosis**, n (%)		
Mood (affective) disorders	Major depressive disorder	81 (33.8)
	Bipolar affective disorder	18 (9.2)
	Dysthymia	2 (0.8)
	*[total]*	*[101 (42.1)]*
Neurotic, stress-related, and somatoform disorders	Social phobia	31 (12.9)
	Obsessive–compulsive disorder	24 (10.0)
	Adjustment disorder	15 (6.3)
	Generalized anxiety disorder	13 (5.4)
	Panic disorder	7 (2.9)
	Agoraphobia	5 (2.1)
	Somatoform disorders	3 (1.3)
	Post-traumatic stress disorder	2 (0.8)
	Specific phobias	2 (0.8)
	*[total]*	*[102 (42.5)]*
Disorders of psychological development	Pervasive developmental disorders	10 (4.2)
	Attention deficit hyperactivity disorder	3 (1.3)
	*[total]*	*[13 (5.4)]*
Schizophrenia, schizotypal, and delusional disorders	Schizophrenia	7 (2.9)
Mental and behavioural disorders due to psychoactive substance use	Mental and behavioural disorders due to use of alcohol	7 (2.9)
Others	Bulimia nervosa	4 (1.7)
	Borderline personality disorder	4 (1.7)
	Insomnia	2 (0.8)
	*[total]*	*[10 (4.2)]*
**Duration of primary diagnosis**, years, mean (SD)		7.6 (7.7)
**Comorbidity**, yes, n (%)		90 (37.5)
**Baseline concurrent psychotropic medications**, yes, n (%)		157 (72.9)
Baseline antidepressant (imipramine equivalent) dose, mg/day, mean (SD)		55.6 (84.5)
Baseline anxiolytic (diazepam equivalent) dose, mg/day, mean (SD)		7.3 (18.2)

**Table 3:** Clinical significance (primary outcome) (n = 217, excluding ongoing cases).

Category	Completer (n = 173)	Intent-to-Treat (n = 217) ^†^
	n (%)	n (%)
**“Recovered”**	74 (42.8)	79 (36.4)
**“Improved”**	34 (19.7)	41 (18.9)
**“No reliable change (unproblematic)”**	36 (20.8)	42 (19.4)
**“No reliable change (problematic)”**	17 (9.8)	30 (13.8)
**“Deteriorated”**	12 (6.9)	25 (11.5)

^†^ For participants who discontinued or dropped out from the therapy, we determined the clinical significance after imputing missing data by carrying forward the last observed scores.

**Table 4:** Changes in secondary outcome measures (n = 217, excluding ongoing cases).

	Completer (n = 173)						Intent-to-Treat (n = 217) ^†^				
	Mean (SD)		t	*p*	ES		Mean (SD)		t	*p*	ES
	Pre	Post					Pre	Post			
**PHQ-9** (n = 173)	12.05 (6.33)	7.07 (5.50)	11.99	<0.001	0.84	**PHQ-9** (n = 217)	12.67 (6.51)	8.44 (6.32)	11.03	<0.001	0.66
**GAD-7** (n = 173)	9.80 (5.51)	5.65 (4.73)	11.01	<0.001	0.88	**GAD-7** (n = 217)	10.24 (5.50)	6.61 (5.33)	10.83	<0.001	0.67
**EQ-5D-5L** (n = 121)	0.67 (0.15)	0.78 (0.16)	−5.28	<0.001	0.71	**EQ-5D-5L** (n = 157)	0.67 (0.14)	0.75 (0.17)	−4.71	<0.001	0.53
**SDS** (n = 55)	9.31 (7.08)	5.76 (6.60)	2.69	0.001	0.51	**SDS** (n = 68)	10.00 (7.32)	6.68 (6.81)	2.72	0.001	0.47

Note: Higher scores on EQ-5D-5L indicate better quality of life, and those on the other measures indicate greater pathology or severity. Abbreviations: ES, effect size (Hedges’ g); EQ-5D-5L, the EuroQoL 5-dimension 5-level; GAD-7, the 7-item Generalized Anxiety Disorder scale; PHQ-9, the 9-item Patients Health Questionnaire; SDS, the Sheehan Disability Scale. ^†^ For participants who discontinued or dropped out from the therapy, missing data were imputed by carrying forward the last observed scores.

**Table 5:** Predictors of positive clinical significance (“Recovered” or “Improved”) following nurse-led individual CBT (univariate logistic regression (n = 217, excluding ongoing cases)).

Predictor Variable	Comparison	Odds Ratio [95% CI]	*p*
**Age**	1-year increment	1.00 [0.98–1.02]	0.85
**Gender**	Female	1 (Reference)	
	Male or gender neutral	0.92 [0.50–1.71]	0.80
**Marital status**	Not having a partner	1 (Reference)	
	Having a partner	0.86 [0.45–1.64]	0.66
**Employment status**	Employed	1 (Reference)	
	Unemployed	1.47 [0.80–2.74]	0.22
**Primary diagnosis**	Mood (affective) disorders	1 (Reference)	
	Neurotic, stress-related, and somatoform disorders	1.35 [0.66–2.74]	0.41
	Disorders of psychological development	1.84 [0.45–7.53]	0.40
	Schizophrenia, schizotypal, and delusional disorders	2.07 [0.21–20.91]	0.54
	Mental and behavioural disorders due to psychoactive substance use	0.69 [0.04–11.49]	0.79
	Others	0.87 [0.35–2.32]	0.82
**Duration of primary diagnosis**	1-year increment	0.99 [0.96–1.03]	0.96
**Comorbidity**	No	1 (Reference)	
	Yes	1.42 [0.73–2.76]	0.30
**Baseline antidepressant (imipramine equivalent) dose**	1mg increment	1.00 [0.99–1.01]	0.24
**Baseline anxiolytic (diazepam equivalent) dose**	1mg increment	1.00 [0.99–1.01]	0.41
**PHQ-9 (pre)**	1-point increment	0.83 [0.78–0.89]	<0.001
**GAD-7 (pre)**	1-point increment	0.78 [0.72–0.85]	<0.001
**Study institution**	Institution I	1 (Reference)	
	Institution II	1.18 [0.34–4.14]	0.80
	Institution III	1.46 [0.43–4.98]	0.55
	Institution IV	0.51 [0.14–1.83]	0.30

Abbreviations: CI, confidence interval; EQ-5D-5L, the EuroQoL 5-dimension 5-level; GAD-7, the 7-item Generalized Anxiety Disorder scale; PHQ-9, the 9-item Patients Health Questionnaire; SDS, the Sheehan Disability Scale.

## Data Availability

Anonymized data from this paper are available for research purposes upon request to the corresponding author. The Ethics Committee at the University of Miyazaki has restricted unlimited public sharing of the data for this study.

## Appendix A. The Relationship between PHQ-9 and GAD-7 Scores before and after Nurse-Led Individual CBT Stratified by Clinical Significance

**Table A1:** Correlation coefficients of PHQ-9 and GAD-7 scores before and after nurse-led individual CBT stratified by clinical significance (n = 217, excluding ongoing cases).

		r
**Pre**	Among participants demonstrating positive clinical significance	0.63
	Among participants demonstrating nonpositive clinical significance	0.81
**Post**	Among participants demonstrating positive clinical significance	0.76
	Among participants demonstrating nonpositive clinical significance	0.85
